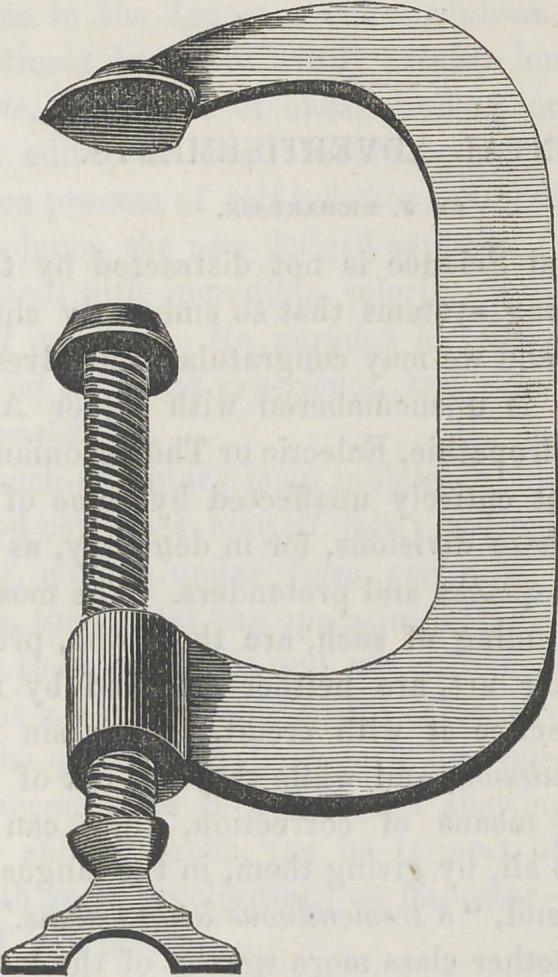# A Clamp

**Published:** 1859-06

**Authors:** J. Taft


### A CLAMP.

The following cut represents a valuable dental laboratory instrument, invented and got up by Dr. Rurras, of N. Y. City.

The form of the instrument will be at once recognized from the figure. It is used for holding down upper plates on the casts, after the palatal portion of the plate has been hammered down, about to its proper form, and both firmly down upon the bench, while the outer portion of the plate is hammered down round the ridge. The part of the clamp having the screw, is placed upon the under side of the bench, and the plate and cast on the bench, under the upper end of the clamp. It is then screwed down firmly, so that it holds the plate in place, while the outer portion is drawn down upon the cast. A roll of paper or some similar substance, should be placed between the plate and clamp, to prevent injuring the plate. Those who have been accustomed to hold down the plates with the fingers upon the casts, while forming them, will fully appreciate the value of this clamp, when they come to use it. T.

## Figures and Tables

**Figure f1:**